# Prevalence of and Factors Associated with Work-Related Musculoskeletal Symptoms in Nursing Assistants Working in Nursing Homes

**DOI:** 10.3390/ijerph15020265

**Published:** 2018-02-04

**Authors:** Kin Cheung, Grace Szeto, Godfrey Kin Bun Lai, Shirley S. Y. Ching

**Affiliations:** 1 School of Nursing, The Hong Kong Polytechnic University, Hong Kong, China; shirley.ching@polyu.edu.hk; 2 Department of Rehabilitation Sciences, The Hong Kong Polytechnic University, Hong Kong, China; grace.szeto@polyu.edu.hk; 3 Hong Kong Association of Occupational Health Nurses, Hong Kong, China; lai1704@yahoo.com

**Keywords:** epidemiology, prevalence, workstyle, working with pain, intention to leave

## Abstract

The prevalence of work-related musculoskeletal symptoms (WRMSs) in different body parts for nursing assistants (NAs) working in nursing homes is currently unknown. The aim of this study was to determine the extent of WRMSs in nursing assistants and the factors associated with them. Four hundred and forty NAs from 52 nursing homes, recruited by convenience sampling, participated in this cross-sectional study in 2014–2015. A valid and reliable study questionnaire was used to collect data. The results of our study found that 88.4% of NAs reported at least one body part with WRMSs. These NAs reported more symptoms in the shoulders than lower back. Adverse workstyle (OR = 1.04, 95% CI = 1.01–1.08) was the only factor associated with WRMSs after adjustment for potential confounders using multivariable logistic regression. This adverse workstyle could be developed because of physical and psychological work demands. Efforts should be directed at integrating “workstyle intervention” into lifestyle physical activity training to this group of healthcare workers.

## 1. Introduction

The demand for nursing home services has increased dramatically and this has become one of the fastest growing industries worldwide, owing to the ageing population and shorter lengths of hospital stays. The occupational health of nursing assistants (NAs) working in nursing homes is paramount because their health affects the quality of care for the residents. NAs in nursing homes are at higher risk of work-related musculoskeletal symptoms (WRMSs) than are their counterparts in hospitals, and other nursing positions such as registered nurses [1,2]. Despite this, little is known about the prevalence of and factors associated with WRMSs in nursing home NAs. Meyer and Muntaner [1] reviewed workers’ compensation databases in the United States and found that nursing home NAs had the highest incidence of work-related injuries as compared with their counterparts in hospitals and home care. In 1987, Jensen [2] reviewed four workers’ compensation agencies from Idaho, North Carolina, New York, and Wisconsin. In the rankings of 24 occupations based on incidence rates of back sprains and strains, NAs ranked first, above construction laborers, garbage collectors, licensed practical nurses, and registered nurses. Despite almost 25 years of prevention and management efforts, NAs continue to have the greatest rate of WRMSs of all occupations. WRMSs in NAs accounted for 53% of the total cases in 2013 in the United States [3]. 

For many years, nurses in many positions, including NAs who work in nursing homes, have reported problems related to WRMSs [4,5,6,7,8]. However, Gao and colleagues [9] found most nursing home studies had focused on registered nurses, despite the observation that NAs provide direct care that involves conventional musculoskeletal risks (i.e., lifting, awkward postures). Furthermore, a recent comprehensive review of the prevalence of musculoskeletal disorders in nursing staff in hospitals, long-term care facilities, and home health care [8] found that the number of studies of nursing staff, including NAs in nursing homes, continues to be limited. The lack of studies in this area may be because NAs often have little power within the healthcare system [10].

Of three studies [10,11,12] conducted in nursing homes, two collected data solely from NAs but focused on the back region only [10,11]. Davis and Kotowski [8] identified research gaps such as limited studies on nursing homes, and a lack of focus on the prevalence of WRMSs in body parts other than the lower back. Furthermore, the contributing factors for WRMSs, such as personal, physical, ergonomic, organizational, or psychosocial, including the role of workstyles or working behaviors [13,14,15], have been identified in the literature. These factors are consistent with the conceptual framework of contributors to musculoskeletal disorders developed by the National Research Council [16]. However, the predominant factors associated with WRMSs might be different among NAs working in hospitals and nursing homes due to the differences of their work environments.

WRMSs are amongst the leading reasons for nursing personnel to leave their jobs [17,18,19]. Graham and Dougherty [10] anticipated that similar patterns of intention-to-leave might be expected for NAs. Reducing WRMSs in nursing homes is both ethical and a prudent business decision; in turn, it will facilitate manpower retention. Thus, the purpose of this study was to estimate the point prevalence of WRMSs in NAs working in Hong Kong nursing homes and identify work-related factors associated with WRMSs.

## 2. Methods

### 2.1. Design and Sampling

The target population of this cross-sectional survey study was NAs working in Hong Kong nursing homes, whose main role was to provide direct resident care, such as turning, feeding, toileting, changing clothes, and changing incontinence pads. NAs in Hong Kong work under a variety of titles, such as care workers, health workers, or other job titles given by the nursing homes. The inclusion criteria were NAs who were Chinese-speaking full-time employees, and who had worked in the nursing home for at least one year. However, those who were pregnant, working permanent night duty, or suffering from any illness with serious medical pathology, such as cancer or systemic inflammatory diseases, or receiving active treatment for musculoskeletal problems that had ended less than one month prior to the study, were excluded. 

Nursing homes were approached by convenience sampling to cover all three regions of Hong Kong: Hong Kong Island, Kowloon Peninsula, and the New Territories [20]. As of September 2017, there was 929 nursing homes including: non-governmental organizations (NGOs) (*n* = 181, 19.48%) and private (*n* = 748, 80.52%) [21]. There were 13,044 health workers and 8,600 care workers in 2013 [22]. After ethical approval was obtained from the Hong Kong Polytechnic University (HSEARS20130301003-05), in 2014–2015, different methods were used to invite the nursing homes to participate in the study. First, a list of their contact information was downloaded from the Social Welfare Department website, and invitation letters were sent by fax to the officers in charge. Second, the officers in charge were contacted by phone or email through the community health nurse networks who provide nursing care to residents in nursing homes. Third, the principal investigator promoted the study in a nursing home association, where some officers in charge met to discuss issues related to nursing homes. Fourth, the participating nursing homes referred other participants. A total of 365 nursing homes were contacted. 

### 2.2. Data Collection Procedure

After access had been approved by the nursing home management, NAs were approached with an explanation of the study and then asked to sign a written consent form acknowledging their agreement to participate. They then completed the questionnaires at work during working hours. Trained research personnel assisted the NAs to complete the questionnaire if there were language difficulties.

### 2.3. Measures 

The questionnaire, “Nursing Assistants Musculoskeletal Symptoms Questionnaire,” was developed for the current study based on a literature review and our previous studies [23,24,25]. Most of the scales used in the questionnaire have been tested for their reliability and validity. The content validity of the questionnaire was evaluated by a panel of four experts in the fields of nursing, occupational health, and physiotherapy, who were invited to rate the item relevancy [26]. The content validity index was 0.99 indicating that the questionnaire has “an appropriate sample of items to represent the construct of interest” (p. 459) [26]. The questionnaire consisted of eight sections measuring the dependent variable (WRMSs) and independent variables of demographic data, physical exposure and exertion rates, ergonomic and manual handling knowledge, ergonomic exposures (EE), psychological and workstyle factors, and intention to leave the job. Following are the descriptions of each section of the questionnaire.

#### 2.3.1. Demographic Data 

Demographic data included age, gender, marital status, education level, self-rated health, exercise patterns, smoking habits, history of surgery, job title, work overtime, lifting/transferring training, years of work experience, body mass index (BMI), and waist to hip ratio.

#### 2.3.2. Self-Reported Musculoskeletal Symptoms 

Self-reported musculoskeletal symptoms were assessed by adopting part of the Standardized Nordic Musculoskeletal Questionnaire (NMQ) about pain, aches, or discomfort in different body parts, including the neck, shoulders, and back, at the time of the survey point prevalence [27]. Furthermore, NAs were asked if those symptoms were work-related or not. Dichotomous responses (yes or no) were used. The NMQ is the most commonly-used symptom survey in occupational health [27,28]. It has been shown to be valid and reliable [24]. This instrument has also been used successfully with nursing personnel in Hong Kong and has shown to be valid and reliable [23,24,25].

#### 2.3.3. Perceived Physical Exertion (PE) 

Participants were asked “How would you rate your physical exertion in performing different activities during your working day?” using a Borg’s rating of PE scale [29] that is a 10-point scale: 0 = “nothing at all”, 1 = “very weak”, 2 = “weak”, 3 = “moderate”, 4 = “somewhat strong”, 5 = “strong”, 7 = “very strong”, and 10 = “very, very strong”. Borg’s rate of PE has been commonly used to measure physical activity intensity level in workplace studies [30,31]. 

#### 2.3.4. Workstyle

Each individual worker’s response to increased work demands [32] was assessed by using the 24-item modified version [25,33] of the Workstyle Short Form, developed by Feuerstein and Nicholas [32]. The only modification was to include all body parts. For instance, the original item of “My hands and arms get tired at work” was modified to “my neck/shoulders/hands/arms/back/hips/thighs/calves /feet get tired at work”. In the present study, the overall Cronbach’s alpha was 0.92. Each item was scored on a five-point Likert scale (0 = almost never, while 4 = almost always), and item scores were summed to derive an overall summary score (24 items) and a summary for each of the five subscales (working through pain with 6 items, social reactivity with 5 items, workplace stressors with 8 items, self-imposed workpace/workload with 3 items, and breaks with 2 items) [33]. The sums of subscales were used for the data analyses. High scores indicated high frequencies of adverse workstyles. 

#### 2.3.5. Ergonomic and Manual Handling Knowledge 

Ergonomic and manual handling knowledge was assessed by a set of 21 questions developed based on the literature review [34,35,36,37]. This set of questions measured knowledge about ergonomic principles, manual handling, and how participants were able to apply this knowledge in practical situations. Our pilot test demonstrated a content validity index of 0.97 and an intra-class correlation coefficient for two-week test-retest reliability of 0.88. The scores for the knowledge were tabulated for data analysis by summing the correct responses.

#### 2.3.6. Perceived Ergonomic Exposures (EEs) 

Perceived ergonomic exposures (EEs) were measured using two subscales (i.e., contribution to WRMSs and encounters frequency). Nine items were developed based on our previous study [25] and literature review [30,34,35,36,37] to evaluate (1) their contribution to participants’ WRMSs (measured by Yes or No responses); and (2) the perceived frequency of encountering these EEs (measured by a four-point Likert scale from never to always). These nine items addressed commonly identified EEs of awkward postures, static postures, repetitive movements, working tools that were poorly maintained, and using tools forcefully. For example, NAs were asked if “sustaining static or awkward upper body/upper limb postures during work (e.g., lean forward or sideways, reaching overhead, reaching down, and extended forward reaches) contributed to their WRMSs” and their frequency of encountering the EE. In this study, the Cronbach’s alpha was 0.82 for EE-contribution to WRMSs; and 0.83 for EE-encounters frequency. The sum of subscales was used for the data analyses. High scores indicated EEs that were identified as related to their WRMSs with highly frequent encounters of these exposures. 

#### 2.3.7. Job Content Questionnaire (JCQ)

A job content questionnaire (JCQ) [38] was used to measure the decision authority (3 items), skill discretion (6 items), psychological job demand (9 items), physical job demand (5 items), supervisor support (4 items), co-worker support (5 items), and residents/their family support (4 items). The JCQ is one of the most frequently used self-administrated questionnaires for measuring the social and psychosocial characteristics of jobs. A four-point Likert scale ranging from strongly disagree to strongly agree was used [39]. Its validity and reliability have been tested in different countries [38]. In the present study, the Cronbach alpha was 0.75. The sum of subscales was used for data analyses.

#### 2.3.8. Other Work-Related Factors

Job satisfaction, job stress, and intention to leave the job were indicated by three individual items, the first two on a four-point Likert scale, and thought of quitting the job with dichotomous responses. The participants were also asked to rate their current work ability from zero (completely no work ability) to ten (completely full ability). In addition, they were asked to assess if their health conditions would allow them to continue to work in the coming two years (no, not sure, and surely yes). 

### 2.4. Data Analysis

All data analyses were conducted using IBM SPSS Statistics version 23 (IBM Corp., Armonk, NY, USA). Descriptive statistics, including means, standard deviations (SDs), frequencies, and percentages, were used to examine all the variables under study. Missing data ranged from 0.22% to 1.36% for the independent variables, and there were no missing data for WRMSs; thus, no replacements were made for the missing data. The musculoskeletal symptoms were considered work related if the NAs indicated the problems were job-related. Bivariate analyses were further employed to compare NAs with and without WRMSs using Chi-square tests and independent samples t-tests. The statistically significant independent variables were screened for multicollinearity. In order to reduce the number of variables in the analysis, the independent variables with *p*-value <0.0001 were retained for the final multivariable logistic regression model. All selected independent variables were included in a single step for analysis. Additionally, a *p*-value <0.05 was considered statistically significant. 

## 3. Results

A total of 522 NAs from 47 nursing homes completed the questionnaires. Eighty-two were excluded because they were pregnant, were not employed full time as NAs, or had worked as NAs for less than one year. Four hundred forty-four questionnaires from 10 NGOs (21.3%) and 37 private (78.7%) nursing homes covering three regions of Hong Kong were used for the data analyses. This ratio of NGO versus private nursing homes was comparable to the actual ratios in Hong Kong of 19.48 and 80.52%, respectively [21]. Furthermore, about 60%–90% of eligible NAs from each nursing home participated and completed the questionnaire. 

### 3.1. Characteristics of the NAs

Table 1 shows the participants’ personal and work characteristics. Most of them were female (98.0%, *n* = 321), married (79.8%, *n* = 351), with a secondary school education (63.6%, *n* = 276), and a mean age of 51.1 years (SD = 9.6). Only 1.4% (*n* = 6) and 1.8% (*n* = 8) smoked or drank alcohol, respectively. Most perceived their general health to be “fair” or “not good” (86.6%, *n* = 381). There was a mean body mass index (BMI) of 24.7 (SD = 3.4). Although 261 NAs (59.3%) performed daily stretching exercises, 55% (*n* = 241) indicated that they “never” exercised at least three times per week with sweating for 30 min or did so “not often”. In addition, only 45 NAs (10.3%) performed muscle-strengthening exercises. 

With regard to work characteristics, while 90.4% (*n* = 397) of NAs had received lifting and transfer training, with a mean number of training hours of 34.6 (SD = 60.1), these participants reported a range of 0.2 to 360 h. The mean years of working as NAs was 10.4 years (SD = 6.4). About a quarter of them (25.5%, *n* = 112) were required to work overtime when necessary. For information, the participants from the subgroup of NGO and private nursing homes were similar on most of the characteristics collected about WRMSs as well as intention to leave.

### 3.2. Prevalence of WRMSs

Table 2 shows the prevalence of WRMSs in the NAs at the time of the survey. Shoulders, lower back, and knees were the most frequent body parts reported. At the time of the survey, 88.4% (*n* = 389) had at least one body part with WRMSs.

### 3.3. Factors Associated with WRMSs by Bivariate Analysis

Table 3 shows the factors associated with at least one body part with WRMSs measured at the time of the survey. NAs with WRMSs tended to be older female workers who perceived their health status to be ‘not good’ and their work as more stressful, and intended to leave their jobs. WRMSs were not related to marital status, education level, previous illnesses, previous surgeries, exercise, different kinds of exercises, type of nursing homes, lifting training, type of work, overtime, consultation with traditional Chinese medicinal doctors, physiotherapists, self-medication for pain, job satisfaction, or expectations that their health conditions would affect their ability to work in the next two years. As well, it is interesting to observe that although the NAs’ work was considered stressful, the majority were satisfied with it [95.3% (*n* = 368) NAs with WRMSs vs. 98.0% (*n* = 50) NAs without WRMSs]. Furthermore, there were no differences between NAs with or without WRMSs in BMI, waist-hip ratio, job task frequency, work ability, workstyle (self-imposed workpace/workload, breaks), and all JCQ subscales except coworker support (see Appendix A).

### 3.4. Predictors of WRMSs by Multivariable Logistic Regression Analysis

Table 4 shows the results of the multivariable logistic regression analyses, with incremental adjustment for potential confounders for WRMSs. Between the groups with WRMSs and without WRMSs, the “without WRMSs” group had fewer NAs (*n* = 51). This “without WRMSs group” was used to estimate the number independent variables for the logistic regression. Using the rule of thumb of 10 subjects per variable, five independent variables with the *p*-value of <0.0001 were selected. Furthermore, after screening for multicollinearity, the total workstyle variable had high correlation with its subscales (ranging from 0.76 to 0.88). Thus, the total workstyle was selected for logistic regression analyses. As a result, job stress, EE (contribution to WRMSs), workstyle (total), age, and gender were used for the analysis. After adjustment for age and gender, EE (contribution to WRMSs), workstyle (total), and job stress still remained statistically significant. However, with further adjustment for all covariates, the only significant factor associated with WRMSs was the adverse workstyle behaviors. According to Nagelkerke’s R^2^, 12.6% of variations in the WRMSs were explained by the model. 

The outcome of WRMSs was measured by the intention to leave. NAs with WRMSs were more likely to have the intention to leave than those without WRMSs (see Table 3). 

## 4. Discussion

The results of the current study confirmed that NAs working in nursing homes experience WRMSs at high rates; 88.4% of them reported pain, aches, or discomfort in one body part at the time of survey. Our study found that the NAs reported more symptoms in the shoulders (53.0%), lower back (41.4%), and knees (37.5%). It is difficult to make comparisons with other studies because most previous studies in nursing homes recruited mixed healthcare workers. For instance, in France, Pelissier and colleagues [40] found the 12-month prevalence of neck, shoulder, elbow, and wrist injuries were 50%, 38%, 10%, and 22%, respectively. However, 58.9% of their study sample involved 58.9% NAs (including nursing auxiliaries, medical and psychological assistants, and social assistants), 27.0% housekeepers, and 14.1% nurses. Another study, conducted in South Korea [12], involved 45.1% NAs and other healthcare workers (e.g., nurses and staff working in kitchen, laundry, and technical posts). They found a 12-month prevalence of shoulder (35.2%), arms (22.8%), knee (20.9%), and lower back injuries (19.8%). Moreover, manually handling residents and changing their clothes were key predictors for shoulder, arm, lower back, and knee WRMSs in the NAs [12]. Nevertheless, unlike hospital settings, the evidence indicates that the lower back was not the most common region for WRMS among NAs in nursing homes, but rather the upper limbs. These differences might be due to the variations in the types of work settings. For example, the percentages of NAs in nursing homes are higher than in hospital settings. Furthermore, lifting facilities in nursing homes might not be as adequate as in hospital settings. The results of our study raise awareness of the need for a more detailed study of the contributing factors for WRMSs in NAs working in nursing homes. 

Research has shown that prominent contributing factors for back disorders are mainly physical loads such as manual handling, heavy physical work, and frequent awkward postures [13]. For upper extremities, the main risk factors are repetition, force, and vibration [13]. However, WRMSs may also be initiated, exacerbated, or maintained when physical risk factors interact with high risk workstyles, such as a tendency to continue to work despite persistent pain [14]. In the current study, after adjustment for demographics, physical, psychosocial, and other independent work factors, adverse workstyle remained a significant factor associated with WRMSs for the NAs. To our knowledge, this is the first study evaluating the effect of adverse workstyles on WRMSs in NAs working in nursing homes. Workstyle is a behavior pattern, defined as how an individual worker performs her/his work to meet the demands of a work task [14]. The Workstyle Model [14] has been used to help explain work-related upper extremity symptoms, particularly in office work populations [14,15,41]. Meijer and colleagues [42] have further proposed that workstyle is a mediating factor for work-related upper extremity pain in general. Our study further found that adverse workstyle was associated with WRMSs across multiple body parts (i.e., from neck to feet). 

The factors associated with WRMSs identified through the use of bivariate analyses in our study could supplement the Workstyle Model to explain the development of WRMSs in NAs in nursing homes. The NAs with WRMSs tended to be the older female workers; they perceived their work to be more stressful and more exposed to ergonomic hazards, and felt that they received less co-worker support. They also had high frequencies of adverse workstyles (such as working through pain, social reactivity, and workplace stressors) and more intended to quit their jobs. Worldwide, it is women who traditionally specialize in activities within the home to support family and take care of young, sick, and old family members [43]. This traditional role supports our study findings that, although the NAs rated the work stressful, they reported satisfaction with their work. Although the NA’s work is tough, dirty, hectic, and unskilled [9,44], they still stay in the workforce; partially because it is a meaningful, caring job [9]. Our local qualitative study [44] in the same population also found that NAs would continue to work even though they had sick leave due to musculoskeletal symptoms, because they did not want co-workers to take up their work. Additionally, reciprocity, karma, and intergenerational benevolence may be rewarding factors. According to an earlier study, Asian NAs, for example, believed that caring for residents would accumulate blessings for their futures (karma), while African or European NAs perceived that helping older people now would result in having young people help them as they age (intergenerational benevolence) [9]. However, over-commitment has been identified as a predictor of neck, shoulder, elbow, and wrist WRMSs in France [40]. Besides, financial needs could be the other reason for NAs to stay in the workforce. NAs are considered as low-wage workers in Hong Kong [45] and the United States [10]. They might not have a choice to work overtime to support themselves [10]. They may not negotiate or bargain for their own occupational health. For instance, they would perceive refusing to perform certain tasks that place them at risk for musculoskeletal problems and reporting WRMSs would put their jobs in jeopardy [10]. Based on the Workstyle Model, we hypothesize that muscle fatigue due to physical ergonomic stressors combined with inadequate work/rest cycles and the adverse workstyle of working through pain may further increase the risk of developing WRMSs. Further studies are needed to investigate the role of workstyle in the development of WRMSs across working populations and various body sites. 

The shortage of NAs is a global issue, and redesign of their work may be essential to their retention. Previously, low back pain and disability have been found to be predictors for leaving work [46]. Our study has found that NAs with WRMSs were more likely to report an intention to quit their jobs. Attention should be paid to their perceived general health. Our study found that more NAs (44.8%) perceived their health “not good” than the Hong Kong female population (33%) or the age group of general population between 45 and 54 (29.4%) [47]. Although the NAs reported satisfaction with their caring work, poor health or function from WRMSs may force them to quit. Multiple approaches to redesign the NAs’ work is necessary to keep these passionate workers in the workforce. For engineering control, work design is one of the components. For instance, totally dependent residents could be arranged in one particular room with a ceiling lift connected to the room and the toilet facilities. However, purchasing a ceiling lift could be a challenge to small and medium-sized enterprises. Other low-cost alternatives or lobbying for the government support could be investigated further. For administrative control, the support from management level is important for allocating resources to reduce WRMSs. The promotion of “No Business Wealth without Workers’ Health” [48] is timely. A Corporate Social Responsibility approach to occupational health and safety can produce real benefits, not only to workers’ health, but also from business and societal perspectives in terms of cost reductions and added values [42]. It is worthwhile to explore strategies to encourage employers to integrate workers’ health as a business strategy to establish a healthy workplace. The needs and barriers of each specific workplace should be evaluated separately and addressed one-by-one. It has been suggested in a systematic review [49] on ergonomic interventions in the healthcare industry that strict technique training is not effective enough to reduce WMSDs significantly. Our study has demonstrated that the association of adverse workstyle with WRMSs. Implementation of adequate work/rest cycle, stretching exercise, reducing work demand, and re-arranging the job tasks might be essential. Furthermore, previous research [50] has reported the integration of “workstyle intervention” into lifestyle physical activity training for office workers, but this approach has not been applied to healthcare workers. 

The findings reported must be considered in light of the design used. The cross-sectional nature of this study precludes any causal inferences; directions or cause-and-effect statements are not possible. However, the associations that were observed may prove helpful in the design of intervention to manage WRMSs in this employee group. The potential of recall bias related to retrospective data collection and selection bias related to the potential of a healthy worker effect should also be considered. Furthermore, excluding those receiving active treatment for musculoskeletal problems would bias the estimate of prevalence. Nevertheless, given the number of participants, wide coverage of the sample in three regions of Hong Kong, and two types of nursing homes, generalizability of these findings to NAs in nursing homes in Hong Kong is likely.

## 5. Conclusions

To our knowledge, this is the first study investigating the prevalence of WRMSs in different body parts and their associations with workstyle behaviors in NAs working in nursing homes. Unlike registered or licensed nurses, nursing-home NAs reported that the highest prevalence of WRMSs was experienced in the shoulders rather than the lower back. Furthermore, the increased likelihood of adverse workstyle was associated with WRMSs, suggesting the importance of work breaks and other measures to recover from high strain work tasks or the development of effective accommodations that reduce excessive workload on the upper extremities and back. Reducing WRMSs might facilitate retention of NAs in nursing homes. As in many other jobs, solutions for such symptoms exist and should be implemented in this group.

## Figures and Tables

**Table 1:** Personal and work characteristics of nursing assistants (NAs) (N = 440).

Characteristics	Number (%)
Gender	N = 440
Female	431 (98.0%)
Male	9 (2.0%)
Marital Status	N = 440
Single	36 (8.2%)
Married	351 (79.8%)
Divorced	22 (5.0%)
Widow	29 (6.6%)
Others	2 (0.5%)
Education Level	N = 434
Primary school	143 (32.9%)
Secondary school	276 (63.6%)
Post-secondary	13 (3.0%)
University	2 (0.5%)
Self-rated Health	N = 440
Very good	2 (0.5%)
Good	57 (13.0%)
Fair	174 (39.5%)
Not good	197 (44.8%)
Very bad	10 (2.3%)
Exercise for 20 min (at least 3 times per week)	N = 438
Never	141 (32.2%)
Not often	100 (22.8%)
Sometimes	108 (24.7%)
Always	89 (20.3%)
Daily Stretching Exercise	N = 440
Yes	261 (59.3%)
No	179 (40.7%)
Daily Muscle Strengthening Exercise	N = 438
Yes	45 (10.3%)
No	393 (89.7%)
Job Title	N = 440
Personal care workers	400 (90.9%)
Health workers	40 (9.1%)
Work Overtime	N = 439
Yes	112 (25.5%)
No	327 (74.5%)
Lifting/Transferring Training	N = 439
Yes	397 (90.4%)
No	42 (9.6%)
	Mean ± SD (Range)
Age	N = 439
	51.1 ± 9.6 (19–69)
Years of Work Experience	N = 440
	10.4 ± 6.4 (1–36)
Physical Exertion (PE)	N = 428
	5.14 ± 2.23 (0–10)
Body Mass Index (BMI)	N = 440
	24.7 ± 3.4 (17.1–40.4)

Percentages may not add up to 100% due to rounding.

**Table 2:** Prevalence of work-related musculoskeletal symptoms (WRMSs) among NAs (N = 440) at the time of survey.

Body Parts with WRMSs	Number (%)
Neck	109 (24.8%)
Shoulders	233 (53.0%)
Elbows/Forearms	121 (27.5%)
Palms/Wrists	96 (21.8%)
Fingers	113 (25.7%)
Upper Back	28 (6.4%)
Lower Back	182 (41.4%)
Hips/Thighs	51 (11.6%)
Knees	165 (37.5%)
Calves	78 (17.7%)
Ankles/Feet	124 (28.2%)
At Least One Body Part with WRMSs	389 (88.4%)

**Table 3:** Factors significantly associated with WRMSs among NAs measured at the time of survey: A bivariate analysis (N = 440).

**Categorical Variables**	**With WRMSs**		**Without WRMSs**		
**Personal Characteristics**	**Number (%)**		**Number (%)**		***p*-Value**
Gender	*n* = 389		*n* = 51		0.04 (χ^2^ 4.24; df 1) (Phi 0.10)
Female	383 (98.5%)		48 (94.1%)		
Male	6 (1.5%)		3 (5.9%)		
Self-Rated Health	*n* = 389		*n* = 51		0.001 (χ^2^ 14.87; df 2) (Phi 0.18)
Good/Very good	44 (11.3%)		15 (29.4%)		
Fair	153 (39.3%)		21 (41.2%)		
Not good/Very bad	192 (49.4%)		15 (29.4%)		
**Work Characteristics**					
Job Stress	*n* = 387		*n* = 51		<0.0001 (χ^2^ 13.58; df 1) (Phi 0.12)
Very stressful/stressful	280 (72.4%)		24 (47.1%)		
Not stressful/very not stressful	107 (27.6%)		27 (52.9%)		
Intention to Leave	n = 386		n = 51		0.013 (χ^2^ 6.18; df 1) (Phi 0.18)
Yes	144 (37.3%)		10 (19.6%)		
No	242 (62.7%)		41 (80.4%)		
**Continuous Variables**		**Mean**	**SD**	***t*-Test (df)**	***p*-Value**
**Personal Characteristics**					
Age	With WRMSs (*n* = 388) Without WRMSs (*n* = 51)	51.48 48.55	9.36 11.09	−2.06 (437)	0.04
**Physical Characteristics**					
Ergonomic Exposures (EE) (contribution to WRMSs)	With WRMSs (*n* = 387) Without WRMSs (*n* = 37)	4.72 3.11	2.63 2.72	−3.55 (422)	<0.0001
EE (encounter frequency)	With WRMSs (*n* = 388) Without WRMSs (*n* = 37)	15.01 11.27	7.93 8.42	−2.72 (423)	0.007
**Work Characteristics**					
JCQ—Coworker Support	With WRMSs (*n* = 387) Without WRMSs (*n* = 51)	10.05 10.61	1.58 1.87	2.32 (436)	0.021
**Workstyle Characteristics**					
Workstyle (total)	With WRMSs (*n* = 388) Without WRMSs (*n* = 51)	34.99 21.69	17.97 13.92	−5.09 (437)	<0.0001
Working Through Pain	With WRMSs (*n* = 388) Without WRMSs (*n* = 51)	9.77 4.25	5.06 3.62	−7.528 (437)	<0.0001
Social Reactivity	With WRMSs (*n* = 388) Without WRMSs (*n* = 51)	4.18 2.51	4.81 3.32	−2.40 (437)	0.017
Workplace Stressors	With WRMSs (*n* = 388) Without WRMSs (*n* = 51)	8.54 5.59	6.30 5.16	−3.20 (437)	0.001

JCQ = Job Content Questionnaire.

**Table 4:** Demographic, ergonomic exposure (EE), workstyle, and job stress associated with WRMSs in NAs working in nursing homes: Results from multivariable logistic regression with incremental adjustment for potential confounders (N = 440).

Variables	Crude Odds Ratio (OR) (95% CI)	OR Adjusted for Age and Gender (95% CI)	OR Adjusted for Age, Gender, and EE (95% CI)	OR Adjusted for Age, Gender, EE, and Workstyle (95% CI)
Age	1.03 (1.00–1.06) *			1.03 (0.99–1.07)
Male (0)	Reference			
Female (1)	0.25 (006–1.04)			0.62 (0.06–7.05)
EE-contribution to WRMSs	1.26 (1.10–1.45) **	1.26 (1.10–1.44) **		1.14 (0.98–1.32)
Workstyle (total)	1.06 (1.04–1.09) ***	1.06 (1.04–1.09) ***	1.05 (1.01–1.08) **	1.04 (1.01–1.08) *
Job Stress				
Not stressful/stressful (1)	Reference			
Very stressful/stressful (0)	2.94 (1.63–5.33) ***	2.88 (1.58–5.25) **	1.89 (0.92–3.86)	1.48 (0.70–3.09)

Bold font: * < 0.05 ** < 0.01 *** < 0.001.

## Appendix A

**Table A1:** Factors not significantly associated with WRMSs among NAs measured at the time of survey: A bivariate analysis (N = 440).

**Categorical Variables**	**With WRMSs**		**Without WRMSs**		
**Personal Characteristics**	**Number (%)**		**Number (%)**		***p*-Value**
Marital Status	*n* = 388		*n* = 51		0.09 (χ^2^ 4.81; df 2) (Phi 0.11)
Single	28 (7.2%)		8 (15.7%)		
Married	312 (80.4%)		39 (76.5%)		
Divorced/widow/others	48 (12.4%)		4 (7.8%)		
Education Level	*n* = 385		*n* = 49		0.49 (χ^2^ 0.48; df 1) (Phi −0.03)
Primary school	129 (33.5%)		14 (28.6%)		
Secondary school or above	256 (66.5%)		35 (71.4%)		
Exercise for 20 min	*n =* 387		*n* = 51		0.72 (χ^2^ 1.35; df 3) (Phi 0.04)
Never	121 (31.3%)		20 (39.2%)		
Not often	89 (23.0%)		11 (21.6%)		
Sometimes	97 (25.1%)		11 (21.6%)		
Always	80 (20.7%)		9 (17.6%)		
Daily Stretching Exercise	*n =* 389		*n* = 51		0.94 (χ^2^ 0.006; df 1) (Phi 0.06)
Yes	231 (59.4%)		30 (58.8%)		
No	158 (40.6%)		21 (41.2%)		
Daily Muscle Strengthening Exercise	*n* = 387		*n* = 51		0.71 (χ^2^ 0.14; df 1) (Phi −0.02)
Yes	39 (10.1%)		6 (11.8%)		
No	348 (89.9%)		45 (88.2%)		
Smoking Habits	*n* = 388		*n* = 51		0.70 (χ^2^ 0.15; df 1) (Phi −0.02)
Yes	5 (1.3%)		1 (2.0%)		
No	383 (98.7%)		50 (98.0%)		
History of Surgery	*n* = 389		*n* = 51		0.07 (χ^2^ 3.41; df 1) (Phi 0.09)
Yes	159 (40.9%)		14 (27.5%)		
No	230 (59.1%)		37 (72.5%)		
**Work Characteristics**					
Job Title	*n* = 389		*n* = 51		0.40 (χ^2^ 0.72; df 1) (Phi 0.04)
Care workers	352 (90.5%)		48 (94.1%)		
Health workers	37 (9.5%)		3 (5.9%)		
Job Satisfaction	*n* = 386		*n* = 51		0.37 (χ^2^ 0.79; df 1) (Phi 0.04)
Very satisfactory/satisfactory	368 (95.3%)		50 (98.0%)		
Very unsatisfactory/unsatisfactory	18 (4.7%)		1 (2.0%)		
Work Overtime	*n* = 388		*n* = 51		0.30 (χ^2^ 1.06; df 1) (Phi 0.05)
Yes	102 (26.3%)		10 (19.6%)		
No	286 (73.7%)		41 (80.4%)		
Lifting/Transferring Training	*n* = 388		*n* = 51		0.66 (χ^2^ 0.20; df 1) (Phi −0.02)
Yes	350 (90.2%)		47 (92.2%)		
No	38 (9.8%)		4 (7.8%)		
Expected to Work Next 2 years	*n* = 379		*n* = 50		0.06 (χ^2^ 2.88; df 1) (Phi −0.08)
Surely yes	276 (72.8%)		42 (84.0%)		
No/not sure	103 (27.2%)		8 (16.0%)		
**Continuous Variables**		**Mean**	**SD**	***t*-Test (df)**	***p*-Value**
**Personal Characteristics**					
BMI	With WRMSs (*n* = 388) Without WRMSs (*n* = 51)	24.59 25.30	3.29 4.40	1.11 (57.56)	0.27
Waist to Hip Ratio	With WRMSs (*n* = 389) Without WRMSs (*n* = 51)	0.87 0.87	0.06 0.07	−0.01 (59.87)	0.99
Ergonomic and Manual Handling Knowledge	With WRMSs (*n* = 387) Without WRMSs (*n* = 51)	76.04 73.20	8.84 12.31	−1.60 (56.99)	0.12
**Work Characteristic**					
Years of Work Experience	With WRMSs (*n* = 389) Without WRMSs (*n* = 51)	10.47 9.78	6.29 7.20	−0.73 (438)	0.47
Physical Exertion (PE)	With WRMSs (*n* = 382) Without WRMSs (*n* = 46)	5.21 4.54	2.21 2.36	−1.92 (426)	0.06
Work Ability	With WRMSs (*n* = 387) Without WRMSs (*n* = 51)	7.80 7.61	1.42 1.81	−0.74 (58.38)	0.46
JCQ—Decision Authority	With WRMSs (*n* = 387) Without WRMSs (*n* = 51)	4.31 4.33	1.26 1.16	0.13 (436)	0.90
JCQ—Skill Discretion	With WRMSs (*n* = 387) Without WRMSs (*n* = 51)	10.64 10.86	2.22 2.18	0.68 (426)	0.49
JCQ—Psychological Job Demand	With WRMSs (*n* = 387) Without WRMSs (*n* = 51)	13.29 12.82	2.72 2.69	−1.17 (436)	0.24
JCQ—Physical Job Demand	With WRMSs (*n* = 387) Without WRMSs (*n* = 51)	7.77 7.41	2.58 2.32	−0.95 (436)	0.34
JCQ—Supervisor Support	With WRMSs (*n* = 387) Without WRMSs (*n* = 51)	7.32 7.47	1.64 1.50	0.62 (436)	0.54
JCQ—Resident Support	With WRMSs (*n* = 387) Without WRMSs (*n* = 51)	7.48 7.73	1.63 1.51	1.02 (436)	0.31
**Workstyle Characteristics**					
Self-Imposed Workpace/Workload	With WRMSs (*n* = 388) Without WRMSs (*n* = 51)	3.78 3.25	2.73 2.61	−1.29 (437)	0.20
Breaks	With WRMSs (*n* = 388) Without WRMSs (*n* = 51)	1.55 1.49	1.62 1.69	−0.26 (437)	0.79
